# Accuracy of risk scales for predicting repeat self-harm and suicide: a multicentre, population-level cohort study using routine clinical data

**DOI:** 10.1186/s12888-018-1693-z

**Published:** 2018-04-25

**Authors:** Sarah Steeg, Leah Quinlivan, Rebecca Nowland, Robert Carroll, Deborah Casey, Caroline Clements, Jayne Cooper, Linda Davies, Duleeka Knipe, Jennifer Ness, Rory C. O’Connor, Keith Hawton, David Gunnell, Nav Kapur

**Affiliations:** 1 Centre for Mental Health and Safety, Manchester Academic Health Science Centre, University of Manchester, Manchester, England; 2 Centre for Suicide Research, University of Oxford Department of Psychiatry, Warneford Hospital, Oxford, England; 3 Centre for Self-harm and Suicide Prevention Research, Derbyshire Healthcare NHS Foundation Trust, Derby, England; 4 Population Health Sciences, Bristol Medical School, University of Bristol, Bristol, England; 5 Institute of Population Health, University of Manchester, Manchester, England; 6 Suicidal Behaviour Research Laboratory, Institute of Health and Wellbeing, University of Glasgow, Glasgow, Scotland; 7 Greater Manchester Mental Health NHS Foundation Trust, Manchester, England

**Keywords:** Self-harm, Suicide, Risk factors, Classification, Outcome

## Abstract

**Background:**

Risk scales are used widely in the management of patients presenting to hospital following self-harm. However, there is evidence that their diagnostic accuracy in predicting repeat self-harm is limited. Their predictive accuracy in population settings, and in identifying those at highest risk of suicide is not known.

**Method:**

We compared the predictive accuracy of the Manchester Self-Harm Rule (MSHR), ReACT Self-Harm Rule (ReACT), SAD PERSONS Scale (SPS) and Modified SAD PERSONS Scale (MSPS) in an unselected sample of patients attending hospital following self-harm. Data on 4000 episodes of self-harm presenting to Emergency Departments (ED) between 2010 and 2012 were obtained from four established monitoring systems in England. Episodes were assigned a risk category for each scale and followed up for 6 months.

**Results:**

The episode-based repeat rate was 28% (1133/4000) and the incidence of suicide was 0.5% (18/3962). The MSHR and ReACT performed with high sensitivity (98% and 94% respectively) and low specificity (15% and 23%). The SPS and the MSPS performed with relatively low sensitivity (24–29% and 9–12% respectively) and high specificity (76–77% and 90%). The area under the curve was 71% for both MSHR and ReACT, 51% for SPS and 49% for MSPS. Differences in predictive accuracy by subgroup were small. The scales were less accurate at predicting suicide than repeat self-harm.

**Conclusions:**

The scales failed to accurately predict repeat self-harm and suicide. The findings support existing clinical guidance not to use risk classification scales alone to determine treatment or predict future risk.

**Electronic supplementary material:**

The online version of this article (10.1186/s12888-018-1693-z) contains supplementary material, which is available to authorized users.

## Background

Emergency Departments (EDs) in England treat over 200,000 presentations for self-harm (intentional self-poisoning or self-injury) each year [1], and the appropriate management of these individuals is important. Clinicians are required to manage a number of risks when treating this population. People who have self-harmed are at greater risk of suicide [2], other causes of premature mortality [3] and comorbid conditions such as alcohol misuse [4] compared to the general population. In efforts to help mental health and non-specialist clinicians manage patients, many hospitals use risk scales, which aim to score or classify patients according to their risk of future self-harm or suicide based on the presence or absence of a specified set of characteristics.

Psychosocial assessment by a mental health clinician is a central component of clinical care and is recommended for each episode of self-harm [5]. These in-depth assessments help clinicians to formulate decisions about follow-up care and reach an informed decision about the risk of further self-harm. There is also evidence that psychosocial assessment may reduce the risk of a further self-harm episode [6, 7]. Formal risk scales are used often by ED and psychiatric clinicians. One study of 32 hospitals across England found that over 20 different risk scales were being used with people who presented after self-harm [8]. This suggests they are in widespread use, with little consensus about which should be used or how well they predict future risk.

A recent systematic review compared the diagnostic accuracy of predicting repeat self-harm of a number of scales [9]. There were no scales that performed well enough to be recommended for use in clinical practice. Another recent meta-analysis pooled positive predictive values from 52 studies of psychological scales predicting repeat self-harm and suicide [10]. The results suggested high-risk classification approaches were unlikely to be clinically useful but also reported high between-study heterogeneity. Another study measured the accuracy of the SAD PERSONS Scale (SPS) for predicting suicide following an emergency department presentation, using administrative data to identify suicide deaths [11]. The study found that the predictive accuracy of the SPS was inadequate to support the use of this risk scale. However, there have been few head-to-head comparisons of risk scales within the same cohort. A comparatively small study (*n* = 483) found that the levels of diagnostic accuracy reached by the five scales investigated meant they had limited clinical utility [12]. The risk scales also performed worse at predicting repeat self-harm than simply asking the clinician or patient to rate their risk. However, the study only recruited individuals receiving a psychosocial assessment from mental health clinician (typically only around 55% of all self-harm patients who present to the ED receive a psychosocial assessment) [6, 13]. This study was too small to consider the outcome of most concern to clinicians – suicide, or to examine diagnostic accuracy of the scales in different subgroups. In the current study we therefore aimed to test four of the risk scales tested in previous research, using data from a large unselected cohort of people presenting to the ED after self-harm.

### Aims of the study

Our specific objectives were:

- To estimate predictive accuracy of the risk scales, using established cut-off points, for identifying a) repeat self-harm and b) suicide
- To test for differences in the predictive accuracy of the scales by groups (age, sex, method of self-harm, professional background of the assessor and self-harm history)

Our hypothesis was that the poor predictive ability of risk scales found in previous smaller studies would hold for this larger unselected hospital cohort and would be replicated for suicide as an outcome.

## Methods

### Data sources

Data were obtained from self-harm cohorts in four separate centres in England. Each centre has an established system to collect data relating to episodes of self-harm presenting to the study EDs. Two of the centres (Bristol and Oxford) are based in the South of England, one in the Midlands (Derby) one in the North (Manchester). The centres collected data from one hospital each with the exception of Manchester, which included three hospitals. The EDs in the study hospitals each had access to psychiatric liaison teams alongside emergency out-of-hours cover from crisis teams or junior psychiatrists.

For all self-harm episodes, basic data were available on method of self-harm (including drugs taken in self-poisoning), time of presentation, age, gender and initial hospital management (for example, admission to a medical bed, referral for a psychiatric assessment). For individuals who were subsequently referred to liaison psychiatry services for a psychosocial assessment, additional data were available including factors precipitating the self-harm, circumstances of the act (such as planning and suicidal intent), social circumstances (such as living arrangements and marital status) and symptoms of depression. For the present study, 1000 consecutive episodes of self-harm, including any repeat episodes by the same individuals, were extracted from each centre’s cohort. The presentations took place over different time periods in each centre but all were between 2010 and 2012. Repeat episodes of self-harm by individuals were included to reflect the real-world ED environment and to be in line with clinical guidance that each episode of self-harm should be assessed comprehensively [5]. In order to preserve the observational nature of the data, no selection/exclusion criteria were applied.

In addition to information from the study hospitals, individuals in three of the centres were matched to Office for National Statistics (ONS) records held by the National Health Service [https://digital.nhs.uk]. For individuals who died, information about the cause of death, verdict and date of death were available. In one of the centres, suicide deaths were identified from the local coroner’s office.

### Scales

We compared the predictive accuracy of the following risk scales: the Manchester Self-Harm Rule (MSHR) [14], the ReACT Self-Harm Rule (ReACT) [15], the SAD PERSONS Scale (SPS) [16] and the Modified SAD PERSONS Scale (MSPS) [17]. The MSHR and ReACT both consist of four items, with a ‘yes’ to at least one of the items resulting in a high risk categorisation and ‘no’ to all items corresponding to low risk. The SPS and the MSPS both include ten items and classify episodes into three risk categories (low, moderate and high). The MSPS also weights four of the items, resulting in maximum score of 14. The items included in each of these scales and the cut-off points for the risk categories are shown in Additional file 1: Table S1. Scales were selected based on an existing systematic review of the diagnostic accuracy of risk scales for predicting self-harm [9]. We included scales that could be re-constructed from routinely recorded information following a self-harm hospital presentation. The scales were also included in a previous study that compared their predictive accuracy when administered by a mental health clinician as part of the psychosocial assessment [12].

Due to the observational nature of the study, some of the individual items for the risk scales were derived from variables related to the core items of interest. For example, the item ‘Stated future intent’ from the two SAD PERSONS Scales was derived in three centres from the Suicide Intent Scale [18] and in another from a binary ‘yes/no’ question about current suicidal plans. In another example, the SPS item ‘Depression or hopelessness’ was available for two of the four sites: in one site it was derived from the presence of either one or two of the items ‘depression’ and ‘hopelessness’ within a list of eight symptoms of depression and in another it was derived from the presence of a diagnosis of affective disorder at the time of presentation.

### Outcome measures

Repeat attendance to the study hospitals with an episode of self-harm within 6 months was our first outcome measure. This was selected because it is a marker for continued distress and need for ongoing clinical care, and has been an outcome in many previous studies [19]. We chose 6 months as the follow-up period as the majority of repeat episodes occur within this time-frame [20]. Our second outcome measure was suicide within 6 months of the self-harm episode. Many studies are insufficiently powered to examine suicide as an outcome, due to low event numbers, but large observational datasets are well suited to this purpose. Individuals who could not be matched to ONS records (*n* = 38/3157) were excluded from the analyses of suicide. Finally, we were interested to see if there were differences in the predictive accuracy of the scales by demographic and clinical sub-groups (age, sex, method of self-harm, self-harm history and according to the professional background of the assessor). These have been identified as factors which may influence the assessment of risk [21].

### Missing data

Data were relatively complete (median levels of completeness for variables 69% (IQR 45% to 92%, range 34% to 100%) with lower completeness for certain variables (e.g. future suicidal intent and depression). Data were more likely to be missing for variables where the individual did not have a psychosocial assessment. For episodes where no assessment took place, there was evidence of bias towards clinicians recording the variable if it was ‘present’ and not if it was ‘absent’. In these instances, variables were imputed as absent, prior to multiple imputation. The potential effect of this would be to underestimate sensitivity and overestimate specificity (defined in Table 1). This included the following items from the SPS/MSPS: depression or hopelessness, organised or serious attempt and rational thinking loss. This approach has been taken in a previous study with similar data [7]. The remaining missing data were largely missing at random, with potential predictors of missingness included in the imputation model. Imputation was conducted using the ‘chained equations’ approach [22] in Stata to generate 50 imputations. Analyses were either conducted on all imputed datasets to generate pooled results, or, where multiple imputation was not compatible with the analytic method, estimates were pooled from m = 1–5 using Rubin’s rules [23].

**Table 1:** Diagnostic accuracy definitions

Sensitivity	The proportion of episodes that were followed by a repeat self-harm episode and were correctly identified by the scale as high risk
Specificity	The proportion of episodes that were not followed by a repeat self-harm episode and were correctly identified by the scale as low risk
Positive predictive value	The probability that the episode identified as high risk by the scale was followed by repeat self-harm
Negative predictive value	The probability that the episode identified as low risk by the scale was not followed by repeat self-harm
Positive likelihood ratio	The increased likelihood of a high-risk scale result in an episode that is followed by repeat self-harm versus one that is not
Negative likelihood ratio	The decreased likelihood of a low-risk scale result in an episode that is followed by repeat self-harm versus one that is not
Diagnostic odds ratio	The odds of a high-risk result in an episode that is followed by repeat self-harm versus one that is not (interpreted the same as an odds ratio)
Receiver operating characteristic (ROC) curve	Graphically shows the overall discrimination ability of a scale to identify episodes that were followed by a repeat self-harm episode compared with those that did not at various cut-off points (plotted as sensitivity versus 1-specifcity). The performance of the scale is indicated by the calculation of the area under the curve (AUC). Higher AUC indicate greater discriminatory power.

In a sensitivity analysis, repeat self-harm outcomes were also examined using a dataset with missing data for scale items coded as not present. This analysis did not include one of the centres due to the unavailability of a scale item used in three of the four scales (living circumstances) in this centre. We also conducted a ‘complete case’ analysis of the MSHR and ReACT by excluding scale items with missing data. Due to the larger number of items in the SPS and the MSPS, too many cases would have been excluded for this approach to be feasible for these scales. For example, while 52% of cases had complete data for at least seven out of ten items on the SPS, only 2% had complete data for all ten scale items. Therefore, excluding all cases with missing data for any one scale item would result in 98% of cases excluded from the analyses.

### Statistical analyses

The predictive accuracy of the scales was examined using the following measures: sensitivity (the proportion of people who repeated self-harm and were correctly identified by the scale as high risk), specificity (the proportion of people who did not repeat self-harm and were correctly identified by the scale as low risk), positive predictive value (the probability that the person identified as high risk went on to repeat self-harm), negative predictive value (the probability that the person identified as low risk did not repeat self-harm), positive likelihood ratio (the increased likelihood of a high risk scale result in a person who repeated self-harm vs. one who did not), negative likelihood ratio (the decreased likelihood of a low risk scale result in a person who repeated self-harm vs. one who did not) and diagnostic odds ratio (the odds of a high risk scale result in a person who repeated self-harm vs. one who did not). Receiver operator characteristic (ROC) curves, which show sensitivity on the *y*-axis and 1 minus specificity on the *x*-axis for all possible scale thresholds were plotted [24]. The area under the curve (AUC), based on the published cut-off points for each scale, was also calculated. The AUC represents the overall proportion of cases correctly predicted by the test; an AUC of 0.5 would suggest the test does not perform any better than chance while an AUC of 1.0 indicates every case is predicted correctly. Chi-square tests were used to examine differences in the AUC between subgroups. Stata V.13.1 and OpenEpi were used for the analyses.

## Results

### Characteristics of the cohort

The 4000 self-harm presentations involved 3157 individuals. 60% (2411) of episodes were by females, 14% (552) by individuals aged 18 or under and 22% (892) aged 45 years or over. The majority of episodes involved self-poisoning with drugs or other substances (81%, 3241) and 19% (759) presentations were by individuals who had self-injured. 55% (2206) of episodes received a psychosocial assessment. In 2759 (69%) of the episodes, individuals had a previous history of self-harm. 28% (1133) of episodes were followed by a repeat episode within 6 months. Amongst the 3962 episodes in which individuals could be followed up for mortality status, the incidence of suicide was 0.5% (*n* = 18).

### Repeat self-harm within six months by scale cut-off points

**Fig. 1:**
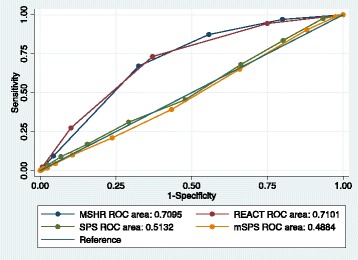
Receiver operator characteristic curves (multiply imputed data, *N* = 4000) for the four scales (MSHR: Manchester Self-Harm Rule; ReACT: ReACT Self-Harm Rule; SPS: SAD PERSONS Scale; MSPS: Modified SAD PERSONS Scale)

For the MSHR and ReACT the majority of the episodes that were followed by a repeat episode in the subsequent 6 months were identified as moderate to high risk (Table 2). The MSHR and ReACT both had high sensitivity for identifying repeat self-harm (98% and 94% respectively) alongside relatively low specificity for identifying those that did not go on to repeat (15% and 23% respectively) (Table 3). The reverse pattern was seen for the SPS and the MSPS: relatively low sensitivity with high specificity resulted in the correct prediction of the majority of episodes that were not followed by repetition of self-harm as low risk. Positive predictive values were similar across the four scales. The positive likelihood ratios for a high risk result on the scales were between 1.2 (MSHR) and 1.3 (MSPS). The overall area under the curve (the proportion of episodes correctly identified by the scales) was highest for the MSHR and ReACT (both 71%) and was approximately equivalent to chance for the SPS (51%) and the MSPS (49%) (Fig. 1).

**Table 2:** Self-harm episodes and repeat self-harm or suicide within 6 months by scale cut-off points (missing data imputed)

Scale	Thresholds	Repeated self-harm (*N* = 1133, 28.3%)	Did not repeat self-harm (*N* = 2867, 71.7%)	Total (*N* = 4000)	Died from suicide (*N* = 18, 0.5%)	Did not die from suicide (*N* = 3944)	Total (*N* = 3962)^a^
Manchester Self-Harm Rule (MSHR)	Low risk (0)	23 (2.0)	435 (15.2)	458 (11.5)	2 (11.1)	450 (11.4)	452 (11.4)
	Moderate/high risk (1+)	1110 (98.0)	2432 (84.8)	3542 (88.6)	16 (88.9)	3494 (88.6)	3510 (88.6)
ReACT Self-Harm Rule (ReACT)	Low risk (0)	63 (5.6)	665 (23.2)	728 (18.2)	4 (22.2)	719 (18.2)	723 (18.2)
	Moderate/high risk (1+)	1070 (94.4)	2202 (76.8)	3272 (81.8)	14 (77.8)	3225 (81.8)	3239 (81.8)
SAD PERSONS (SPS)	Low risk (0–4)	781 (68.9)	2029 (70.1)	2810 (70.3)	12 (66.7)	2766 (70.1)	2778 (70.1)
	Moderate risk (5–6)	251 (22.2)	642 (22.4)	893 (22.3)	4 (22.2)	885 (22.4)	889 (22.4)
	High risk (7–10)	101 (8.9)	196 (6.8)	297 (7.4)	2 (11.1)	293 (7.4)	295 (7.4)
Modified SAD PERSONS (MSPS)	Low risk (0–5)	1020 (90.0)	2561 (89.3)	3581 (89.5)	15 (83.3)	3532 (89.6)	3547 (89.5)
	Moderate risk (6–8)	99 (8.7)	276 (9.6)	375 (9.4)	3 (16.7)	369 (9.4)	372 (9.4)
	High risk (> 8)	14 (1.2)	30 (1.0)	44 (1.1)	0 (0)	43 (1.1)	43 (1.1)

^a^38 individuals were lost to mortality follow-up

**Table 3:** Measures of diagnostic accuracy^a^ for repeat self-harm with 95% confidence intervals, *N* = 4000, m (missing data imputation) =1–5

Scale	Thresholds	Sens %	Spec %	PPV %	NPV %	LR+	LR-	DOR
MSHR	Low risk (0) vs. moderate/high risk (1+)	98 (97, 99)	15 (14, 17)	31 (30, 33)	95 (93, 97)	1.155 (1.154, 1.156)	0.13 (0.12, 0.15)	8.6 (5.6, 13.2)
ReACT	Low risk (0) vs. moderate/high risk (1+)	94 (93, 96)	23 (22, 25)	33 (31, 34)	91 (89, 93)	1.23 (1.228, 1.231)	0.240 (0.230, 0.250)	5.1 (3.9, 6.7)
SPS	Low risk (0–4) vs. moderate risk (5–6)	24 (22, 27)	76 (74, 78)	28 (25, 31)	72 (71, 74)	1.01 (0.98, 1.04)	0.996 (0.993, 0.999	1.0 (0.9, 1.2)
	Moderate risk (5–6) vs. high risk (7–10)	29 (24, 34)	77 (74, 79)	34 (29, 40)	72 (69, 75)	1.23 (1.16, 1.30)	0.931 (0.923, 0.939)	1.3 (1.0, 1.7)
MSPS	Low risk (0–5) vs. moderate risk (6–8)	9 (7, 11)	90 (89, 91)	26 (22, 31)	72 (70, 73)	0.9 (0.7, 1.1)	1.01 (1.008, 1.012)	0.9 (0.7, 1.1)
	Moderate risk (6–8) vs. high risk (> 8)	12 (7, 20)	90 (86, 93)	32 (19, 48)	74 (69, 78)	1.3 (0.4, 3.6)	0.97 (0.95, 0.99)	1.3 (0.7, 2.6)

^a^*Sens* sensitivity, *Spec* specificity, *PPV* positive predictive value, *NPV* negative predictive value, *LR+* positive likelihood ratio, *LR-* negative likelihood ratio, *DOR* diagnostic odds ratio

### Suicide within six months by scale cut-off points

Only two people who died by suicide (11% of all suicides) were identified as low risk by the MSHR and four (22%) by ReACT (Table 2). However two thirds of the suicides (12/18) were categorised as low risk by the SPS and over 80% (15/18) by the MSPS. There were no suicide deaths identified as high risk by the MSPS (Table 2). The MSHR and ReACT had relatively high sensitivity for predicting suicide, while the SPS and the MSPS had higher specificity (Table 4).

**Table 4:** Measures of diagnostic accuracy^a^ for suicide with 95% confidence intervals, *N* = 3962, m = 1–5

Scale	Thresholds	Sens %	Spec %	PPV %	NPV %	LR+	LR-	DOR
MSHR	Low risk (0) vs. moderate/high risk (1+)	89 (65, 99)	11 (10, 12)	0.5 (0.3, 0.7)	99.6 (98.4, 99.9)	1.003 (0.99, 1.02)	0.97 (0.35, 2.68)	1.03 (0.24, 4.50)
ReACT	Low risk (0) vs. moderate/high risk (1+)	78 (52, 94)	18 (17, 19)	0.4 (0.2, 0.7)	99.5 (98.6, 99.9)	0.95 (0.91, 0.99)	1.2 (0.74, 2.0)	0.8 (0.3, 2.4)
SPS	Low risk (0–4) vs. moderate risk (5–6)	25 (7, 52)	76 (74, 77)	0.5 (0.1, 1.2)	99.6 (99.3, 99.8)	1.0 (0.2, 4.5)	1.0 (0.8, 1.2	1.0 (0.4, 3.2)
	Moderate risk (5–6) vs. high risk (7–10)	33 (4, 77)	75 (73, 78)	0.7 (0.1, 2.4)	99.6 (98.9, 99.9)	1.3 (0.2, 9.6)	0.9 (0.5, 1.5)	1.5 (0.3, 8.3)
MSPS	Low risk (0–5) vs. moderate risk (6–8)	17 (4, 41)	91 (90, 91)	0.8 (0.2, 2.3)	99.6 (99.3, 99.8)	1.8 (0.1, 4.5)	0.9 (0.8, 1.0)	1.9 (0.6, 6.6)
	Moderate risk (6–8) vs. high risk (> 8)	0 (0, 71)	90 (86, 92)	0 (0, 8)	99.2 (97.7, 99.8)	-^b^	–	–

^a^*Sens* sensitivity, *Spec* specificity, *PPV* positive predictive value, *NPV* negative predictive value, *LR+* positive likelihood ratio, *LR-* negative likelihood ratio, *DOR* diagnostic odds ratio

^b^No suicides were identified as high risk by the MSPS

### Differences by subgroups

There were few differences in overall predictive accuracy (AUC) by subgroups (Table 5). There was higher AUC for episodes assessed by a psychiatrist for both the SPS (area under the curve 0.61, *p* = 0.003) and the MSPS (0.57, *p* = 0.001) than those assessed by a mental health nurse, by another profession or not assessed. The ReACT risk scale performed worse amongst episodes involving individuals with no prior episode of self-harm (0.60) compared to individuals with a history of self-harm (0.67, *p* = .0.02). However, the absolute differences between subgroups were small (Table 5).

**Table 5:** Area under the curve (AUC) for repeat self-harm by subgroups, with 95% confidence intervals, *N* = 4000, m = 1–5

	MSHR	ReACT	SPS	MSPS
Overall AUC (95% CI)	0.71 (0.69, 0.73)	0.71 (0.70, 0.73)	0.51 (0.48, 0.54)	0.49 (0.47, 0.51)
Male (*n* = 1589)	0.69 (0.67, 0.72)	0.69 (0.66, 0.72)	0.54 (0.51, 0.57)	0.52 (0.49, 0.55)
Female (*n* = 2411)	0.72 (0.70, 0.74)	0.73 (0.70, 0.75)	0.51 (0.49, 0.54)	0.49 (0.46, 0.52)
Aged < 19 (*n* = 552)	0.73 (0.68, 0.78)	0.73 (0.68, 0.78)	0.47 (0.42, 0.52)	0.48 (0.42, 0.53)
Aged = > 19 to 44 (*n* = 2556)	0.71 (0.69, 0.73)	0.71 (0.69, 0.73)	0.51 (0.49, 0.54)	0.48 (0.46, 0.50)
Aged 45+ (*n* = 892)	0.71 (0.67, 0.74)	0.71 (0.68, 0.75)	0.54 (0.49, 0.58)	0.51 (0.47, 0.56)
Main method of harm				
Self-poison (*n* = 3241)	0.71 (0.69, 0.73)	0.72 (0.70, 0.74)	0.53 (0.51, 0.55)	0.50 (0.48, 0.52)
Self-injury (*n* = 759)	0.70 (0.67, 0.74)	0.67 (0.64, 0.71)	0.47 (0.43, 0.51)	0.47 (0.42, 0.51)
Assessed by:				
Psychiatrist (*n* = 638)	0.67 (0.63, 0.71)	0.64 (0.60, 0.69)	**0.61 (0.56, 0.66)**	**0.57 (0.52, 0.62)**
Mental health nurse (*n* = 1273)	0.69 (0.66, 0.72)	0.72 (0.69, 0.75)	0.51 (0.47, 0.54)	0.47 (0.44, 0.51)
Other (*n* = 295)	0.67 (0.61, 0.74)	0.71 (0.64, 0.77)	0.50 (0.42, 0.57)	0.44 (0.37, 0.51)
History of self-harm (*n* = 2759)	0.63 (0.61, 0.66)	**0.67 (0.65, 0.69)**	0.45 (0.42, 0.48)	0.44 (0.41, 0.46)
No history of self-harm (*n* = 1241)	0.64 (0.59, 0.69)	0.60 (0.53, 0.67)	0.49 (0.42, 0.55)	0.46 (0.40, 0.52)

Bold text denotes statistically significant (*p* < 0.05) difference between groups

### Sensitivity analysis

In sensitivity analysis with missing items imputed as not present, the MSHR and ReACT again performed with relatively high sensitivity and low specificity, though sensitivities were lower and specificities higher, due to more individuals being rated as low risk (Additional file 1: Table S2). The measures of predictive accuracy of the SPS and MSPS were broadly similar to those in the main analyses. When we included only those cases with complete data for all scale items for the MSHR and ReACT scales, sensitivity was similar but specificity was lower. For the MSHR, 30.4% (981/3228) of episodes were followed by repetition of self-harm, sensitivity was 99.2% and specificity was 7.4%. For ReACT, the repeat rate was 27.2% (668/2459), sensitivity was 94.3% and specificity was 20.0%.

## Discussion

### Main findings

The MSHR and ReACT performed with high sensitivity but low specificity for prediction of repeat self-harm. This resulted in a large proportion of episodes that were not followed by repetition being placed in the higher risk category. The overall area under the curve for these scales was fair. The SPS and the MSPS had lower sensitivity and higher specificity, resulting in the majority of episodes that resulted in repetition being identified as low risk. The SPS and the MSPS were no better than chance in terms of overall predictive accuracy. The SPS and the MSPS identified the majority of suicide deaths as low risk.

### Strengths and limitations

Some scale items were not available across all sites. To avoid over-estimating the prevalence of risk factors, we assumed the item was not present if data were missing. Furthermore, if there was evidence that the prevalence of a scale item was over-estimated in non-assessed episodes, missing items were imputed as absent for all non-assessed episodes before multiple imputation. The effect of this on the measures of predictive accuracy would be to underestimate sensitivity (due to fewer meeting the criteria for ‘high risk’) and overestimate specificity (due to more meeting the criteria for ‘low risk’). When the performance of the MSHR and the ReACT scales, the two best performing scales in this study, was tested on cases with complete data for all scales items, measures of sensitivity were similar and specificities were lower. This suggests our imputation approach did not lead to over-estimation of the performance of these two scales.

The data for this study were obtained from observational self-harm cohorts with data extracted from hospital records and clinical notes. It was necessary to use proxy variables for certain scale items where an exact corresponding variable was not available, which is a limitation of this study. In addition, for some scale items, corresponding variables could be recorded differently according to the study centre. For example, suicidal intent was derived from the Suicide Intent Scale in three centres and from a binary variable in another centre. This would have had most impact on the SAD PERSONS Scales, due to the higher number of scale items. However, the measures of diagnostic accuracy are in line with a previous study which showed the area under the curve to be no better than chance for the SPS [17]. Furthermore, this novel approach resulted in a more representative, real-world sample of self-harm episodes and their management.

A potential source of bias in this study could have arisen if percieved risk influenced subsequent clinical management, which in turn may have been associated with risks of repeat self-harm and suicide. This would have led to bias in the measures of predictive accuracy.

### Comparison to previous research

A previous study used a selected sample with clinicians administering the risk scales following referral to psychiatric services [12]. The conclusion from that study was that the diagnostic accuracy of the scales was too modest for them to be of clinical use. The measures of diagnostic accuracy in the present study are comparable, suggesting that risk scales following self-harm are also unsuitable for the wider population of all those who present to hospital following self-harm, not just those seen by mental health clinicians. While the previous study recruited patients after they had been referred to liaison psychiatry for assessment, the present study also included non-referred episodes, typically just under half of all episodes [13, 25]. The present study also suggests that the risk scales are unsuitable for predicting risk of future suicide among individuals presenting with self-harm. A meta-analysis of risk scales used for predicting suicidal behaviour also found that predictive ability of risk scales was insufficient to be used to determine treatment allocation [10]. The present study addresses the high level of heterogeneity found in the meta-analysis, and reaches similar conclusions.

A comparatively small study of psychiatric inpatients recently reported a modular multi-informant approach, resulting in promising levels of accuracy for predicting further suicidal behaviour [26]. Machine learning techniques utilising medical databases are also becoming more common in the pursuit of accurate detection of suicide risk [27]. These are potential areas for further research.

### Clinical implications

This study adds to the evidence that scales, particularly the widely used SAD PERSONS Scales, are not suitable for predicting repeat episodes of self-harm or future suicide. Their overall performance as measured by the AUC did not surpass a ‘fair’ level of prediction, defined as between 0.7 and 0.8 [28], likelihood ratios had weak predictive ability [29] and performance did not exceed that of clinicians’ ratings (measured at 0.74) found in an earlier study [12].

There is evidence that risk classification scales remain in widespread use despite growing evidence about their poor predictive abilities [8, 10]. It is possible that clinicians welcome the structure they offer or the prompts for factors to consider, such as social isolation, in their overall formulation of a follow-up plan. The use of risk scales may act as ‘aide memoires’ for less experienced clinicians and may help in eliciting the relevant information, provided the patient’s narrative is not lost [30]. Carter and colleagues also suggest that there should be a focus on modifiable risk factors, such as hopelessness, as part of a needs-based assessment [10]. This would help focus the assessment on a person’s situation and how best to help manage it [31]. There is evidence that assessment itself may be beneficial at reducing the risk of a repeat self-harm episode [6, 7], with patients valuing a positive therapeutic alliance that promotes hope and encouragement [32].

The SPS and MSPS performed slightly better for episodes assessed by a psychiatrist, though the performance was still below those of the ReACT and MSHR scales. Episodes assessed by a psychiatrist were more likely to involve individuals with a history of self-harm but not currently receiving treatment. The episodes by individuals assessed by psychiatrists were also more likely to be rated as moderate or high risk than low risk on both the SPS and the MSPS compared to episodes assessed by mental health nurses. In addition, the overall predictive accuracy of the ReACT scale was lower for episodes where individuals had no history of self-harm. These individuals were less likely to repeat self-harm, and the specificity was lower (16%), suggesting the incorrect identification of episodes that did not repeat resulted in reduced overall performance. However, the difference in predictive accuracy between these groups was small and is unlikely to be of major clinical importance.

## Conclusion

The findings of this study support existing clinical guidance, which suggests scales that classify patients into risk categories should not be used alone to allocate treatment or predict future risk of further self-harm or suicide. There was no evidence to support the use of risk scales with particular subgroups of patients. While scales with high sensitivity may have some clinical use when considered alongside a comprehensive assessment [15], they are not suitable for the purpose of prediction. Given that scales continue to be widely used by clinicians, future studies could consider if they could be combined with other approaches to increase their effectiveness. For example, randomised controlled trials could combine structured risk assessment with other aspects of care, such as comprehensive follow-up planning or building therapeutic alliance. A study carried out recently in the United States found that screening in the ED combined with a brief intervention consisting of a number of components (including safety planning and follow-up telephone calls), was associated with reductions in repeat suicide attempts and overall number of repeat attempts [33]. Further studies of this kind could help to determine aspects of risk scales that might be useful in the management of self-harm.

## Additional file

Additional file 1: Supplementary tables: additional information about the risk scales and their items. Description of material: **Table S1.** Risk scales tested in the predicting risk of repeat self-harm cohort study, **Table S2.** Risk scale items and corresponding variables from the self-harm cohort studies. (DOCX 23 kb)

## Abbreviations

AUC: Area under the curve
ED: Emergency Department
MSHR: Manchester Self-Harm Rule
MSPS: Modified SAD PERSONS Scale
ONS: Office for National Statistics
ReACT: ReACT Self-Harm Rule
ROC: Receiver operating characteristic
SPS: SAD PERSONS Scale
