# Supplementary material for: Accuracy of risk scales for predicting repeat self-harm and suicide: a multicentre, population-level cohort study using routine clinical data
Source: BMC Psychiatry. 2018 Apr 25;18:113. doi: 10.1186/s12888-018-1693-z (PMC5921289; doi:10.1186/s12888-018-1693-z)
Supplement: Supplementary file 1 — Supplementary tables: additional information about the risk scales and their items. Description of material: Table S1. Risk scales tested in the predicting risk of repeat self-harm cohort study, Table S2. Risk scale items and corresponding variables from the self-harm cohort studies. (DOCX 23 kb) [file 12888_2018_1693_MOESM1_ESM.docx]

Supplementary tables: additional information about the risk scales and their items

Table S1: Risk scales tested in the predicting risk of repeat self-harm cohort study

| **Scale** | **Number of items** | **Scale items** | **Risk categories (published cut-off points)** |
| --- | --- | --- | --- |
| Manchester Self-Harm Rule | 4 | • Any history of self-harm  • Prior psychiatric treatment  • Benzodiazepines used in self-poisoning  • Current psychiatric treatment | The presence of any one indicates moderate/high risk |
| ReACT Self-Harm Rule | 4 | • Recent self-harm (past year)  • Cutting as method of harm   - Lives alone or homeless   • Current psychiatric treatment | The presence of any one indicates moderate/high risk |
| The SAD PERSONS scale | 10 | • Male sex  • Older age  • Depression  • Previous suicide attempt  • Excess alcohol or  substance use  • Rational thinking loss  • Social supports lacking  • Organised plan  • No spouse  • Sickness | 3 categories of risk:  0-4 = low  5-6 = moderate  7-10 = high |
| The Modified SAD PERSONS scale | 10 | • Male sex  • >19<45  • Depression or hopelessness  • Previous suicidal attempts  or psychiatric care  • Excessive ethanol or drug  use  • Rational thinking loss  • Single, widowed or  divorced  • Organised or serious  attempt  • No social support  • Stated future intent | 3 categories of risk:  0-5 = low  6-8 = moderate  9-14 = high |

Table S2: Risk scale items and corresponding variables from the self-harm cohort studies

| **Scale** | **Scale items** | **Corresponding variables**  **Centre 1** | **Centre 2** | **Centre 3** | **Centre 4** |
| --- | --- | --- | --- | --- | --- |
| Manchester Self-Harm Rule | • Any history of self-harm  • Prior psychiatric treatment  • Benzodiazepines used in self-poisoning  • Current psychiatric treatment | - Any history of self-harm - Prior psychiatric treatment - Benzodiazepines used in self-poisoning - Current psychiatric treatment | - Any history of self-harm - Prior psychiatric treatment - Benzodiazepines used in self-poisoning - Current psychiatric treatment | - Any history of self-harm - Prior psychiatric treatment - Benzodiazepines used in self-poisoning - Current psychiatric treatment | - Any history of self-harm - Prior psychiatric treatment - Benzodiazepines used in self-poisoning - Current psychiatric treatment |
| ReACT Self-Harm Rule | Addition scale items not included in SAD PERSONS Scale  • Recent self-harm (past year)  • Cutting as method of harm   - Lives alone or homeless | - Self-harm within the past 12 months - Cutting as method of harm - Living status on day of self-harm | - Self-harm within the past 12 months - Cutting as method of harm - Not available | - Self-harm within the past 12 months - Cutting as method of harm - Living status | - Self-harm within the past 12 months - Cutting as method of harm - Marital status |
| The SAD PERSONS scale | • Male sex  • Older age  • Depression  • Previous suicide attempt  • Excess alcohol or  substance use  • Rational thinking loss  • Social supports lacking  • Organised plan  • No spouse  • Sickness | - Male sex - Age in years - Psychiatric diagnosis (affective disorder) - History of self-harm - Alcohol or drug misuse - Not available - Not available - Not available - Marital status - Presence of a physical disorder | - Male sex - Age in years - Not available - History of self-harm - Referred to alcohol/drug addiction services - Not available - Not available - Beck Suicide Intent Scale (total score) - Marital status - Not available | - Male sex   • Age in years  • Feels depressed  • Previous suicide attempt  • Current alcohol or  substance misuse  • Current visual/audial hallucinations or delusions  • Not available  • Premeditated act  • Marital status  • Physical health problem as precipitant to the act | - Male sex   • Age in years  • Not available  • Previous suicide attempt  • Current alcohol or  substance misuse  • Not available  • Social isolation as a precipitant to the act  • Beck Suicide Intent Scale (total score)  • Marital status  • Physical health problem as precipitant to the act |
| The Modified SAD PERSONS scale | Addition scale items not included in SAD PERSONS Scale    • Depression or hopelessness  • Stated future intent | - Depression only (from psychiatric diagnosis) - Beck Suicide Intent Scale (total score) | - Not available - Beck Suicide Intent Scale (total score) | - Depression or hopelessness   • Stated wanted to die | - Not available - Beck Suicide Intent Scale (total score) |
